# Supplementary material for: Are carbon emissions trading and green financial instruments synergistic? -Comprehensive quantitative research based on content analysis
Source: PLoS One. 2024 Mar 7;19(3):e0298601. doi: 10.1371/journal.pone.0298601 (PMC10919601; doi:10.1371/journal.pone.0298601)
Supplement: S1 Data — (ZIP) [file pone.0298601.s001.zip › Data/地级市Z_F工作报告.zip!/╔╜╢1⁄2/╝├─╧╩╨/└┤╫╘╣1⁄2╓┌║┼í╛┬φ┐╦╩2╛▌═°í┐_2010.docx]

各位代表：

　　我代表市人民政府向大会报告工作，请予审议，并请市政协委员和其他列席人员提出意见。

一、2009年工作回顾

　　过去的一年，是新世纪以来我市经济社会发展最困难、各项任务最繁重的一年。在中共济南市委的坚强领导下，全市以科学发展观为统领，坚决落实党中央、国务院和省委、省政府扩内需、促消费、保增长决策部署，深入贯彻胡锦涛总书记视察山东重要讲话精神，积极应对国际金融危机的冲击和考验，科学务实，积极作为，全力做好“保增长、保民生、保稳定、保全运”各项工作，经济社会实现了平稳较快发展。预计全市完成生产总值3350亿元，增长12%左右；地方财政一般预算收入210.2亿元，增长13%；全社会固定资产投资1655.4亿元，增长20.3%。完成了市十四届人大二次会议确定的目标任务。

　　（一）保增长取得积极成效。及时出台实施3600亿元投资计划、八大产业调整振兴规划以及促进中小企业、成长型企业发展的一系列政策措施，开展“项目推进年”和帮扶企业活动，全市经济逐季回升向好。坚持把扩内需作为着力点，争取中央新增投资项目200个、资金4.7亿元，省调控资金24.1亿元，其中前三批178个项目实现开工率、资金配套率和问题整改率“三个百分之百”。“项目推进年”233个重大项目完成投资579亿元。工业投入完成541.8亿元，增长24.8%，吉利轿车、青年商务车等一批项目投产见效。投资结构进一步优化，农业、高新技术、现代服务业、基础设施投资分别占全部投资的3.7%、8.8%、42.4%、26%。努力增强消费对经济增长的拉动作用，实现社会消费品零售总额1615亿元，增长19%。大力培育旅游、汽车等消费热点，深入推进万村千乡市场工程。启动67个棚户区片区改造，建设安置房210万平方米，新建农村住房6.4万户，改造危房1.1万户。商品房销售面积439.8万平方米，增长18.5%。家电下乡销售收入增长3.2倍。努力稳定外需市场，实现进出口总额55亿美元，其中出口30亿美元，降幅逐步收窄。实际到账外资9.7亿美元，增长12.1%。应对金融危机措施迅速到位，有力促进了经济稳定回升。

　　（二）转型升级步伐加快。大力推进结构调整和发展方式转变，加快构建以服务经济为主的产业体系。三次产业比例由5.8：44.1：50.1调整为5.5：43.5：51。农业、建筑业、规模以上工业和服务业增加值分别实现185亿元、220亿元、1154亿元和1710亿元，增长5%、17.4%、11.2%和13%。非公有制经济比重达到42.5%，提高1.5个百分点。大力发展现代服务业，金融业增加值增长19.6%，软件、旅游、会展、物流等产业营业收入分别增长33.6%、22.1%、26.4%、13.7%，现代服务业占服务业比重达到43.7%。引进浙商银行等9家金融机构，全市金融机构达到156家。新增上市公司2家，市域上市公司、股票总数分别为26家和28只，数量居全省之首。金融机构本外币存贷余额分别为6422.7亿元和6200.6亿元，增长25.2%和27.9%。高新技术企业达209家，高新技术产业产值占规模以上工业的比重达39.5%，提高2个百分点。高新区和8个省级开发区工业产值占全市比重达31.2%，成为新的增长点。“数字泉城”建设取得突破，部、省、市共建中国软件名城试点正式启动，信息化惠民、强政、兴业重点工程加快推进。自主创新能力增强，取得重大科技成果330项，国家级、省级、市级创新型企业分别达到2家、8家、50家。企业技术中心187家、工程技术研究中心132家，分别增加23家和31家，中国重汽集团获准组建国家级工程技术研究中心。国家级火炬计划特色产业基地达7家，占全省的30%。制定《济南市知识产权战略纲要》，专利申请量及授权量、发明专利申请量及授权量继续居全省首位，新认定中国驰名商标4件、省著名商标48件。电网建设加快推进，电力输入能力提高55%。节能减排成效显著，成为全国首批城市节能与新能源汽车试点示范城市，淘汰立窑水泥熟料产能89万吨，万元生产总值能耗和二氧化硫、化学需氧量排放量进一步下降。

　　（三）城市功能形象全面提升。抓住筹办第十一届全运会的历史机遇，全面提升规划建设管理水平。实施迎全运80项城市建设重点工程和90项环境综合整治项目，建成以“东荷西柳”为代表的奥体中心等一批全运场馆工程，东部新区初具规模。加快以西客站片区为中心的西部新城建设，西部地区发展取得重要进展。着力打造泉城特色标志区，大明湖扩建改造及护城河西线通航工程完成。加快交通、市政等基础设施建设，二环东路高架路、奥体中路、经十西路等一批重点道路建成通车，舜耕路南段、顺河西街、玉函路等市区主要道路整修改造相继竣工。坚持公交优先战略，新增公交车415部，其中新能源环保车100部，开通6条大运量快速公交线路，成为国内第一个形成快速公交网络的城市。小清河综合治理一期工程及工商河、东泺河等市区河道治理竣工，城市防汛工程建设加快推进。扩大水、电、气、暖管网覆盖面，城市燃气气化率、集中供热普及率分别达到97.8%、49%，提高0.2个、3个百分点。加强燕翅山等地质灾害治理，完成58座破损山体整治，新增城市绿地608公顷，绿化覆盖率达36.4%，提高0.8个百分点。机扫保洁率达62%，提高7个百分点。垃圾日处理能力由2600吨提高到2900吨，污水处理率达85%以上。实施重点废气污染源限期治理工程和扬尘污染防治措施，市区空气质量逐步改善。持续推进城市环境综合整治，有效化解一大批城市管理难点问题，绿化、美化、净化、亮化成效显著，城市形象全面提升。成功举办第七届国际园林花卉博览会，23个国家、86个城市参展，建成108个展园，创造了参展城市、展园数量历届之最。

　　（四）新农村建设成效显著。全面落实强农惠农政策，扎实推进新农村建设“十大行动”。全年粮食总产289.5万吨，连续7年实现丰收。都市型、城郊型农业初具规模，集中打造章丘大葱、商河瓦西冬瓜等15个特色品牌基地和月亮湾、长香源等30个都市农业园区，推出胜源梨花节、仁风富硒西瓜节等6大类观光休闲农业精品项目。规模以上农业企业和农民专业合作社分别达300家和2011家，增加30家和912家。畜禽养殖规模化、标准化水平进一步提高。累计认证无公害、绿色、有机食品369个，推广新技术41项，农产品质量安全体系不断完善。实施南水北调济南段、腊山分洪等重点水利工程，完成48座病险小型水库除险加固，治理润玉泉、八里峪等18条小流域。村村通油（水泥）路率、通客车率、通自来水率和通有线电视率分别达99.3%、99.5%、95%和100%，完成283个电气化乡（镇）、村建设，农村用电量增加20%。防治美国白蛾、秸秆综合利用和禁烧工作成效显著，重大动物疫病防控扎实有效。落实“四项补贴”资金5亿元，增长31.6%。政策性农业保险试点工作成效明显。集体林权制度改革试点顺利推进，完成荒山绿化4700公顷。森林覆盖率达28.9%，提高1.1个百分点。修编完成土地利用总体规划并通过国土资源部审查。编制完成《南部山区保护发展规划》、38个城镇总体规划和500个新农村规划，重点扶持10个小城镇建设。土地整理复垦开发5113公顷，土地流转积极稳妥推进。落实加快商河发展的18项扶持政策成效显著。实施农村扶贫开发项目30个，带动10.8万低收入农民增收。

　　（五）民生和社会事业全面加强。去年承诺的为民办9件实事全面完成。人民生活进一步改善，城市居民人均可支配收入22722元，增长9.2%；农民人均纯收入7770元，增长8.2%。居民消费品价格指数保持在100.33。就业形势保持稳定，安置就业再就业14.8万人，转移农村富余劳动力18.8万人次。基本养老、医疗、失业、工伤、生育保险参保范围进一步扩大。城镇居民医疗保险参保率达90%。城乡低保和农村五保供养标准进一步提高，68家敬老院建成达标。4489户家庭享受到廉租住房补贴和实物配租，增加1155户。棚户区改造动迁居民15.3万人，顺河街等片区8000户居民陆续回迁。推动农村住房建设与危房改造，105个村庄整体搬迁项目启动。全部免除农村义务教育阶段学生作业本费和寄宿生住宿费，农村中小学“211”工程和校舍升级改造加快推进。启动医药卫生体制改革，实施卫生强基工程，社区卫生服务中心（站）发展到222所，基本实现城区居民全覆盖。完成2377所村卫生室建设改造，新农合参合率达98.8%。人口自然增长率控制在5‰以内。加快市属文化单位体制改革步伐，启动文化产业“3810”工程创建活动，完成60个乡镇综合文化站及800个农家书屋建设，全市公共博物馆免费开放。创作推出《天下泉城》、《北方有佳人》等优秀电视作品，儿童剧《我和我的影子》获全国优秀演出奖。我市运动员在十一届全运会获金牌17枚，创历史最高水平。推动群众体育进农村、进社区、进校园、进机关，全民健身设施达299.4万平方米，连续四届被评为“全国群众体育先进单位”。

　　（六）和谐济南建设扎实推进。以开展迎国庆60周年、迎全运系列活动为契机，加强和谐社会建设各项工作。扎实开展深入学习实践科学发展观活动，围绕解难题、察民情、办实事，解决了一批制约科学发展、群众反映突出的热点难点问题。开展“迎全运、讲文明、树新风”活动，组织实施十大文明行动，市民文明素质和城市文明程度大幅提升，涌现出泉城义工、公交温馨服务、贴心民政等一批精神文明建设品牌和历下区、市中区等一批国家、省和谐社区建设示范单位。依法、民主、科学决策机制进一步完善，法治城市、法治县（市）区创建全面推进，“法律六进”工作不断深化。深入推进平安济南建设，加强社会治安综合治理，严厉打击各类刑事犯罪，开展交通秩序、暂住人口、网络环境等专项整治，群众安全感明显增强。加大基层矛盾纠纷排查调处力度，“大接访”和“信访积案化解年”活动取得明显成效。健全应急管理机制，稳妥做好甲型流感等重大疫情疫病防控工作。推进重点行业、领域安全生产治理行动，安全生产形势基本稳定。工商、质监、食品药品监管等部门加强食品药品源头、生产、流通、使用等环节综合监管，市场秩序进一步规范。积极推进双拥共建工作，国防教育、征兵工作、国防后备力量和人民防空建设不断加强。荣获全国民族团结进步模范集体和全国防震减灾综合评比第一名，对口支援北川灾后恢复重建工作进展顺利。新闻出版、广播电视、社会科学、档案史志、外事侨务、对台工作、妇女儿童、老龄、残疾人、慈善事业和气象等工作取得新成绩。

　　各位代表，过去的一年，我们经受了严峻考验，取得了来之不易的成绩。特别是，在党中央、国务院和省委、省政府的坚强领导下，我市圆满完成了第十一届全国运动会主赛区的各项任务。广大干部群众顽强拼搏、攻坚克难，以强烈的责任意识，建设了一流的比赛设施，提供了高效文明的服务保障和富有创意的赛事组织。动员11.4万名志愿者开展全运服务，全运村接待运动员、裁判员和媒体记者1万多人，实现零失误、零差错的目标。出动警力36万人次，组织1.8万名安保力量，确保了火炬传递、开闭幕式和700多场赛事圆满成功，为举办富有特色、平安祥和、人民满意的全运会作出了突出贡献，赢得了中央领导、省委省政府、参赛人员和社会各界的充分肯定和高度赞誉，也收获了丰厚的物质成果和宝贵的精神财富，市民自信心、城市美誉度全面提升。在这里，我代表市政府，向在各行各业辛勤劳动的全体市民，向给予政府工作大力支持的各级人大代表、政协委员，各民主党派、工商联和无党派人士，向关心帮助省会工作的驻济人民解放军、武警官兵，向为济南发展作出贡献的港澳台同胞、海外侨胞、国内外朋友们，向来济兴业的广大投资者和外来务工人员，表示崇高的敬意和衷心的感谢！

　　各位代表，在总结成绩的同时，我们更清醒地看到，济南的发展还存在一些问题：经济回升的基础还不稳固，外需萎缩局面未有大的改变，民间投资增幅较低。经济结构性矛盾仍较突出，产业结构调整相对滞后，部分工业行业和重点企业尚未摆脱困境，节能减排和污染治理的压力仍然很大。城市承载功能不足，基础设施和综合服务功能难以满足更高层次发展的需要。民生保障体系和社会事业发展水平与群众需求差距较大，就业、住房、医疗、教育、文化等方面存在不少突出问题。政府自身建设有待加强，腐败现象和损害群众利益的问题时有发生。对这些问题，我们将在今后工作中进一步强化措施，努力加以解决。

二、2010年经济社会发展的总体要求和主要目标

　　2010年是实施“十一五”规划的最后一年，也是转方式调结构、确保经济平稳较快发展的关键一年。从国际经济发展看，金融危机的深层次影响依然存在，全球范围内的产业分工和结构调整进程加快。从国内看，在促进经济平稳较快发展的前提下，转变经济发展方式、调整优化产业结构成为主旋律，提高经济增长的质量、效益和可持续性成为主要目标。从济南发展看，转型发展、创新发展成为今后长期的发展主线，省会经济发展进入以创新为先导、高端产业为驱动的新阶段。做好今年的工作，对于巩固经济回升基础、启动实施“十二五”规划，都有十分重要的意义。

　　2010年全市经济社会发展的总体要求是：全面贯彻党的十七大和十七届三中、四中全会精神，坚持以科学发展观为统领，按照中央、省、市经济工作会议的决策部署，着眼于推进济南跨越提升、转型升级和全面突破，拓展城市发展空间，打造现代产业体系，全力推进转方式、调结构、促增长、惠民生、保稳定的重点工作，加快建设宜业宜居的现代化省会城市和区域性中心城市，努力实现转型发展、创新发展、和谐发展。

　　2010年经济社会发展的主要预期目标是：生产总值增长11%，地方财政收入增长12%，全社会固定资产投资增长20%，社会消费品零售总额增长17%。服务业增加值增长13%，规模以上工业增加值增长12%，高新技术产业产值比重提高2个百分点。外贸出口和利用外资实现恢复性增长。城市居民人均可支配收入和农民人均纯收入增长8%，居民消费价格总水平涨幅控制在3%左右，城镇登记失业率控制在4%以内。万元生产总值能耗和二氧化硫、化学需氧量排放量全面完成“十一五”规划目标。人口自然增长率控制在5‰以内。各项指标都力争在“十一五”规划的基础上完成得更好一些。

　　实现上述目标，任务艰巨，责任重大。市政府在今年的工作指导上，必须把握好以下要点：一是抓住发展的机遇期、关键期，拓展城市发展空间，把重点区域带动与培植产业发展优势结合起来，增强集聚辐射能力，努力增创省会发展新优势。二是增强发展的创新性和可持续性，加快自主创新，推动战略性新兴产业发展，打好转方式、调结构攻坚战，形成高端高质高效的现代产业体系。三是努力保障和改善民生，把促进经济发展与改善公共服务结合起来，发展社会事业，改善人居环境，把惠民生的各项事情办实办好。四是进一步解放思想，提升境界，继续发扬筹办全运会形成的良好精神风貌和工作作风，努力在新的起点上创造新的业绩。

三、2010年经济社会发展的主要任务

　　（一）努力保持经济平稳较快发展。把握好国家宏观调控政策导向，在稳定扩大投资规模的同时，促进消费、投资、出口协调拉动经济增长。一是发挥投资对结构调整的导向作用。抓好支撑带动作用强的重大项目，重点推进在建项目，严格按照国家产业政策新上项目。加快重汽产业园、鲁能产业园、北车风电工业园等一批工业项目建设。把西客站片区、魏家庄片区等一批城市综合体作为新区开发、老城提升、服务业发展的龙头，高起点规划建设。推进城市文化中心等一批重大文化设施规划建设，加快轨道交通、沿黄湿地等重大项目前期工作。二是加强项目投资建设管理。继续开展“项目推进年”活动，积极争取国家和省支持资金、银行信贷资金。加强项目审计监察，有效化解债务风险。坚持节约集约用地，防止低水平重复建设和盲目扩张。完善重点项目分级联系制度，健全重大项目推进机制和绿色通道制度。抓好项目筛选储备，重点增加交通、能源、农业水利、社会民生、高新技术、现代服务业等领域投资。三是拓展培育消费增长点。落实好家电汽车下乡、大型农机具购置补贴和家电汽车以旧换新、小排量车辆购置税减征等促进消费政策，支持居民自住和改善性住房消费，拓展便民消费和服务性消费领域。增强居民特别是低收入群体消费能力，完善最低工资制度，提高群众消费预期。继续实施万村千乡市场工程和下乡产品流通网络工程，建设600个标准化农家店，增设39处10万平方米便民菜市场和250个社区便民网点，改造提升一批特色商业街。进一步规范市场经济秩序，加强价格调控监管，健全产品质量检测体系，切实改善消费环境。

　　（二）着力建设高端高质高效现代工业体系。把转方式、调结构与推进主导产业高端化、新兴产业规模化、传统产业品牌化结合起来，抓好增量带动结构优化升级，力争工业投资增长20%以上。一是推进工业振兴。落实中央和省、市扶持工业发展的财税政策，培育战略性新兴产业，加快光伏发电产业化、半导体照明新材料、重大新药创制平台和国家级生物医药产业基地建设，推进新能源、新材料、生物医药三大新兴产业规模化。加大高新技术和先进制造业投入，力争北车风电产业园等24个项目上半年竣工，加大鲁能电工电气等32个重大项目推进力度，高新技术和先进制造业投资占工业投资的85%以上。交通装备、电子信息、机械制造三大主导产业主营业务收入分别超过1000亿元。冶金钢铁、石油化工、食品饮料、新型建材等传统产业做优品牌，实现优化提升，努力打造以绿色经济、循环经济为特征的现代工业产业体系。二是增强园区集聚配套能力。省级以上园区和重点企业要率先成为转方式、调结构的主力军，完善基础设施，创新体制机制，促进产业集聚。高新区充分发挥政策优势和产业优势，加快国家信息通信国际创新园、山东信息通讯研究院和齐鲁软件园等重点产业园区建设，努力打造新的经济增长极和高新技术产业、先进制造业创新基地，8个省级园区也要实现较大突破。三是优化企业发展环境。加强对企业服务，加大增值税转型、技改贴息、金融信贷、企业减负等惠企政策落实力度，加强重点行业和企业监测跟踪，稳定煤电油运供应，积极组织产销衔接和各类银企对接活动，深入实施中小企业技术创新、小企业培育、中小企业成长和特色产业提升“四项计划”。四是全面提高自主创新能力。以进入首批国家创新型试点城市为动力，完善提升“六大体系”，创新产学研合作机制，推进高校院所自主创新计划，鼓励科技成果进园区转化。实施一批重大科技专项，力争在节能减排、光电风电、新材料、高性能服务器等方面取得一批技术成果，推进千万亿次计算平台落户济南。办好第四届中国生物产业大会、第五届信博会和第六届科技展交会。各级企业技术中心、工程技术研究中心达350家以上，培育100家创新型企业和300家成长型科技企业，推动社会研发和技改投入较快增长。实施品牌带动战略，加强知识产权使用和保护，培育发展一批自主品牌、优势产品和骨干企业。强化创新人才队伍建设，开展“5150”工程，引进扶持一批海内外领军人才及创业团队。五是全面完成节能减排任务。继续抓好百户耗能企业和十大节能工程，严控“两高”行业低水平重复建设，完成小火电关停任务，实施早期脱硫设施再提高工程，创建扬尘污染控制区，加强机动车排气污染等大气环境综合防治。

　　（三）充分发挥现代服务业的引领带动作用。把促进服务业发展作为扩大消费的重要增长点和结构调整的着力点，拓宽领域、增强功能、扩大增量、提升层次，增创发展新优势。一是强化生产性服务业的支撑作用。做强区域性金融中心，以魏家庄片区改造和西客站片区建设为契机，推进金融商务中心区和后台服务区建设，完善多元化、多层次的金融服务体系。构建区域性信息中心，加强信息传输基础设施建设，推广3G网络应用，加大软件产业扶持力度，加快齐鲁外包城建设，在国家级电子信息制造业基地、中国软件名城和“数字泉城”创建上取得新进展。建设区域性现代物流中心，用好我市作为全国性物流节点城市的优惠政策，扶持第三方物流企业做大做强，推进物流信息平台建设。整合会展资源，打造特色品牌，形成规模优势和集聚效应。二是改造提升生活性服务业。按照打造战略性支柱产业的要求，加快文化创意及动漫游戏、旅游会展、体育健身、休闲娱乐产业发展，推进国家级动漫产业基地和文化园区建设，扶持一批具有泉城特色和影响力的文化创意项目。壮大旅游市场主体，培育大型旅游集团，构建大旅游产业。发展电子商务、连锁经营等新型业态和经营模式，增强服务业的辐射带动力。挖掘生活性服务业吸纳就业的潜力，扶持发展特色餐饮服务业，统筹规划一批社区商业、养老托幼、家政服务等社区服务业项目，推进社区服务规范化和网络化，满足群众生活和就业需求。加强房地产市场调节和引导，增加普通商品住房供给。三是加快服务业载体建设。建设历下、市中等7个重点集聚区和盖家沟国际物流园、济南国际会展中心等30个重点园区，培育100家重点企业和100个重点品牌。加快总部经济、楼宇经济和税源经济发展，筹划、引进和建设一批服务业项目，发展财务、法律、咨询等中介组织，逐步形成种类齐全、功能完善的现代服务体系。

　　（四）加快推进城乡一体化发展。把“三农”工作作为重中之重，继续推进新农村建设“十大行动”，促进城乡统筹协调发展。一是积极发展都市型、城郊型现代农业。调整优化农业产业结构，确保粮食安全，做强畜牧业、做优蔬菜业、做大林果花卉业，积极发展特色农业、设施农业、生态农业、观光休闲农业和现代渔业，培植壮大鲍家芹菜、商南彩椒等30个特色品牌基地和都市农业园区，新发展规模以上农业企业35家，扶持农民专业合作社示范社50家。二是促进农民持续增收。搞好农村多种经营，加快发展农村二、三产业，加大实用技术培训力度，为农民创造更多就业和增收机会。强化农业产业、经营、科技、标准等支撑体系建设，确保农畜产品质量安全，加快无规定动物疫病区建设。三是强化基础设施建设。加快南水北调济南段、东湖水库和腊山分洪工程建设，完成卧虎山等3座大中型水库和36座小型水库除险加固，实施田山、邢家渡、胡家岸等灌区续建配套和节水改造工程，加大小流域综合治理力度。抓好荒山绿化、退耕还林、平原绿化、绿色通道等林业重点工程，完成造林8000公顷，完善农村气象监测预报服务体系，加强黄河堤防建设。四是加快推进小城镇建设。用好建设用地置换和城乡建设用地增减挂钩政策，搞好土地综合整治项目试点，推进新型农村社区建设。实施城镇化建设提升工程，促进供水、供电、公交、通信、污水和垃圾处理网络化体系建设，扎实推进农村小康环保行动计划。实施道路畅通提升工程，加快市、县（市）区、乡镇、村路网建设。解决20万农民饮水安全问题，农村自来水入户率达80%以上。五是提升县域经济实力。统筹城乡发展，加强分类指导，立足产业实际和长远发展，因地制宜规划产业布局，推进市区工业和基础设施向县域延伸，鼓励经济强县（区）增强发展质量与效益，继续支持商河等远郊县加快发展，实现城镇化、工业化和农村现代化协调发展。

　　（五）全面提升城市规划建设管理水平。切实把城市建设作为促发展、惠民生的战略措施抓紧抓好，着眼于增强承载功能，进一步拓展空间、完善配套、加强管理，在打造宜业宜居的现代化城市上实现新的突破。一是坚持科学规划引领。高起点编制各类专业专项规划，进一步完善规划体系。提升东部新区、西部新城、小清河区域和老城区改造的规划策划，深化《济南市城市综合体发展布局规划》，完善《泉城特色标志区规划》。严格实施新一轮土地利用总体规划。二是拓展城市发展空间。奥体文博片区重点强化生活居住和公共服务设施建设，加快发展高新技术产业、先进制造业和金融服务、商务会展、科教研发、体育休闲等产业，带动210平方公里东部新区加快发展。西客站片区围绕“齐鲁新门户、泉城新商埠、城市新中心”的目标定位，加快6平方公里核心区、26平方公里中心区建设，带动120平方公里西部新城快速发展。小清河两岸重点搞好综合整治和连片开发，逐步将沿岸120平方公里打造成宜业宜居的新城区。三是改造提升老城区。提高土地利用率，推进棚户区、城中村和危旧简易楼群改造，把老城区打造成功能布局合理、空间利用高效、服务经济发达的现代化城市中心。四是加快城市基础设施建设。无论新区拓展还是老城提升，都要把基础设施建设放在突出位置，适度超前，支撑发展。基础设施建设投入不低于全社会固定资产投资的25%。优化城区路网结构，实施二环西路等道路建设改造和张庄路等50条道路大、中修工程，规范地下管网设施。做好省会城际铁路建设前期工作，支持京沪高铁西客站、石济客专等重点项目建设，开工建设济商高速公路，实施国道220线平阴段、104线黄河北段改造。启动1000千伏济南特高压输变电工程建设。推进鹊华、玉清水厂升级改造，加快第二垃圾处理厂和水质净化四厂、中水处理站建设，强化污水处理厂运营监管，实现城市污水全收集全处理。实施防汛能力提升工程，完善排水体系，加强雨水收集设施建设。加快莲花山、西客站热源厂建设和供暖管网建设改造，完善城区天然气输配系统。优先发展大公交体系，搞好大型公交停车场站和换乘中心建设，推广智能交通系统，缓解交通拥堵。推进柳行河、龙脊河等4条干线河道、8条支线河道的截污改造工程。五是推进生态建设。完善“增雨、置采、控流、补源、节水”综合措施，继续做好保泉各项工作。做靓泉城特色标志区，深化明府城改造规划策划，启动百花洲片区改造试点，推进东护城河通航改造工程。严格落实南部山区保护与发展规划，加大饮用水源地保护力度，抓好城区绿化，加快西郊森林公园建设，建成一批区级公园、街头游园和绿地广场，整治完成第二批50座破损山体。六是加强城市精细化管理。巩固提升迎全运城市环境综合整治成果，加大建筑渣土、违法违章建设、违法户外经营和城市脏乱差回潮等重点领域和薄弱环节整治力度。继续推进城市管理重心下移，切实把区街工作重点转到城市管理、社区服务上来。整合完善数字化城管系统，构建管理与执法高效统一的“大城管”格局。

　　（六）继续深化改革扩大开放。深化改革破解发展难题，扩大开放赢得发展机遇，为经济社会又好又快发展注入动力和活力。一是深化国有企业改革。加快国有企业改制重组，通过改制上市、股权多元化改造等有效形式，优化国有经济布局，积极推进12户困难企业改革。健全国有资产监管体系，完善国资监管风险管控机制和惠民生保稳定长效机制，实现国有资产保值增值。二是大力发展民营经济。加快推进垄断性行业体制改革和市政公用事业改革，放宽市场准入，鼓励和促进民间资本以多种形式进入基础产业、基础设施、社会事业、金融服务等领域。三是推动资源型产品价格改革。开展用电大户与企业直接交易试点，推进部分基础产品价格改革。四是稳妥推进农村改革。深化农村综合配套改革，增强乡镇政府社会管理和公共服务职能。健全土地承包经营权流转市场，鼓励适度规模经营，推动集体林权制度和水管体制改革。五是抓好财税金融体制改革。落实好省直管县财政体制改革试点，开展扩权强镇和供销合作社改革试点，支持、规范面向“三农”的村镇银行、专业贷款公司和小额贷款组织发展。积极探索投融资体制改革。六是深化社会事业改革。推进文化事业和文化产业体制改革，完成市属艺术院团转企改革任务，创新社会事业发展模式，鼓励社会资本兴办社会事业，优化社会事业资源配置。深化医药卫生体制改革，着力抓好基本医疗保障制度等5项改革，努力为群众提供安全、有效、方便、价廉的医疗卫生服务。

　　大力提高对外开放水平。切实把外经贸工作重点转到拓市场、保份额、促平衡上来。一是扎实推进市场多元化战略。稳定传统市场，深度开拓南美、非洲、俄罗斯等新兴市场。抓住与台湾直航的有利时机，加强对台经贸合作，引导企业用好在线广交会等对外贸易平台。优化出口产品结构，扩大机电、高新技术产品出口，扶持有出口潜力的中小企业建设自主营销网络。优化出口通关流程，推进出口加工区保税区建设。二是扩大招商引资规模。做好项目策划包装，组织实施产业招商。坚持以我为主、择优选资，引导外资投向战略性新兴产业、高新技术产业和高端制造研发环节。积极争取世界银行、亚洲开发银行优惠贷款，做好国外贷款项目筛选储备工作。三是大力发展服务外包。加大政策扶持、法规保障、人才培养引进力度，扩大日韩外包业务，拓展欧美市场，推进“中国服务外包示范市”建设，力争软件与信息服务外包收入翻番。四是大力实施“走出去”战略，鼓励企业对外投资、承包工程和外派劳务，收购国际品牌、营销网络、研发中心和矿产资源。实现对外承包工程、劳务合作合同额20亿美元，营业额12亿美元。做好外事、侨务工作，积极拓展对外交流渠道和领域。

　　（七）努力保障和改善民生。把改善民生、发展社会事业作为扩内需和调结构的重点，切实解决涉及群众切身利益的问题。完成为民办12件实事。一是努力扩大就业再就业。继续实施积极的就业政策，挖掘政策投资和大项目带动就业的潜力，大力发展就业型产业，做好援企稳岗工作。健全创业服务体系，抓好就业见习基地和创业孵化园区建设。鼓励自谋职业、自主创业，建立服务、培训、维权三位一体的农村劳动力输出工作机制，重点做好零就业家庭、残疾人、低保对象、破产企业职工、就业困难的普通高校毕业生就业工作。城镇新增就业11万人，农村劳动力转移就业16万人次，援助就业困难人员1万人，“双零家庭”保持动态消零。二是加快完善社会保障体系。扩大社会保障覆盖范围，城镇基本养老、医疗、失业、工伤、生育保险参保人数分别达到144万、243万、84.9万、111万、68万，做好中断缴费人员社会保险接续，实现城镇无收入老年居民养老保险应保尽保，搞好新农保试点。提高企业退休人员养老金、城乡低保、农村五保供养标准，加强大病救助和困难家庭临时救助，加快公益性社会福利设施建设，大力发展慈善事业。三是改善群众住房条件。抓好保障性安居工程，加快廉租住房、实物配租房和棚改安置房建设，基本完成集中连片棚户区改造任务。加快城中村改造、农村住房建设和危房改造，新建农村住房7万户，改造危房1.5万户。进一步提高住房公积金覆盖率和使用率，惠及更多职工群众。四是推进教育均衡发展。完善政府助学体系，实现困难学生助学金全覆盖，确保适龄青少年全面接受义务教育。加强新建改建小区配套学校建设，加大中小学校房屋改造力度，推进盲、聋残疾儿童集中免费就读，解决好困难家庭和外来务工人员子女上学问题。以实施“1610”工程为主线，推进实训基地建设，增强职业教育服务功能。五是提高居民健康保障水平。加强城乡基层卫生队伍及服务能力建设，新农合筹资保障标准提高到120元。落实国家基本药物制度，保证群众基本用药的可及性和安全性。加强城乡公共卫生体系建设，增强突发公共卫生事件处置能力。加强人口和计划生育工作，统筹解决人口问题，保持低生育水平持续稳定。六是加快构建公共文化服务体系。以筹办第十届中国艺术节为契机，建设一批标志性公共文化设施，推出一批文艺创作精品。加快社区文化设施建设，按时完成农家书屋建设任务。七是探索全运会后体育场馆综合利用和管理运作方式，搞好运营维护，促进体育事业发展。

　　（八）维护社会和谐稳定。注重夯实社会和谐的基础，营造平安稳定的社会环境。一是高度重视平安创建工作。大力推进法治城市建设，加强社会治安综合治理，依法打击各类犯罪活动，完善领导干部定期接访、下访制度，畅通信访渠道，做好矛盾纠纷排查化解工作，不断完善社会治安防控体系。扩大法律援助覆盖面，保障弱势群体的合法权益。二是狠抓基层基础建设。探索社区管理服务体制改革，推广“五位一体”管理模式，解决好社区服务设施建设问题。提高村（居）民自治水平，搞好第十届村委会换届选举工作，支持企事业单位加强民主管理。抓好民族宗教工作，进一步促进民族团结。三是高度重视公共安全。加强应急体系建设，有效预防和处置各类突发事件，做好地震和极端天气等自然灾害的预警防范，强化公共避灾场所和人防工程建设管理。继续做好援川工作。加强安全生产、食品药品安全监管，做好甲型流感疫情防控工作，确保人民群众生命健康安全。四是大力加强精神文明建设。以“争做泉城文明人、争创全国文明城”为主题，推进新一轮文明城市创建工作。实施市民文明素质提升工程，创造更好的精神文明建设活动品牌，引导市民从自我做起，提高思想道德和文明素质，做好社会志愿服务工作，推进精神文明创建常态化、科学化、制度化。扎实做好双拥共建工作，争创双拥模范城“七连冠”。

四、努力推进服务型政府建设

　　新的形势和使命要求我们以发展转型为契机，创新政府工作，提高行政效能，增强引领科学发展的能力，加快推进服务型政府建设。

　　（一）加快政府职能转变。全面完成机构改革任务，进一步理顺工作机制，创新行政管理体制。深化行政审批制度改革，清理审批事项，优化审批流程，确保政府机构改革中要求取消、下放、转移的事项落实到位。建立公共服务、社会管理、公共安全及行政管理标准体系，规范政府行为，提高行政效率。以综合考评为抓手，加大对行政不作为和乱作为的问责力度，促进政府服务创新创优。逐步理顺公共资源管理体制，加快公共资源交易中心建设。组织编制“十二五”发展规划，提高对转型发展的推动力和调控力。

　　（二）提高工作透明度和公信力。坚持把科学民主决策作为行政基本制度，规范决策程序，健全重大事项集体研究、专家咨询、社会公示与听证、决策评估等制度，完善各类公开办事制度。加强电子政务建设，健全网络平台的信息公开、网上办事、调研咨询和督查落实等运行机制，办好12345市民服务热线。依法保障群众知情权、参与权、表达权和监督权。提高依法行政能力，规范行政自由裁量权，加强权力运行监控，开展行政复议委员会试点工作，探索推进相对集中行政复议权制度。自觉接受人大及其常委会监督，接受政协民主监督，认真听取民主党派、工商联、无党派人士和各人民团体的意见，接受新闻舆论和社会公众监督。

　　（三）扎实推进惩治和预防腐败体系建设。深化反腐倡廉教育，严格遵守廉洁从政各项纪律规定，完善防治腐败的制度体系，从腐败多发和易发环节入手，严查征地拆迁、企业改制、安全生产、环境保护、重点工程、食品药品安全、社保基金和住房公积金管理等方面的案件，加强对扩内需、促增长项目和资金的跟踪审计监察。坚持领导干部经济责任审计制度，运用现代科技手段预防腐败，强化对重点岗位、领域的监督。

　　（四）发扬求真务实的工作作风。通过作风转变促进各项任务落实，用工作实效检验作风建设成果。坚持说实话、办实事、求实效，扑下身子、埋头苦干。完善目标管理责任制，逐级落实经济社会发展目标，研究确定工作措施，破解发展难题，强化督察督办，抓好工作落实。全体政府工作人员特别是领导干部要树立正确的价值观、政绩观，切实增强宗旨意识、公仆意识、节俭意识和服务意识，坚持问政于民、问需于民、问计于民，关心群众疾苦，倾听群众呼声，解决群众合理诉求，多办顺民意、解民忧、惠民生的实事，切实改善民生、造福百姓。

　　各位代表，形势催人奋进，任务光荣艰巨。让我们更加紧密地团结在以胡锦涛同志为总书记的党中央周围，在中共济南市委的坚强领导下，解放思想，提升境界，抢抓机遇，团结拼搏，为“维护省城稳定，发展省会经济，建设美丽泉城”作出新的更大贡献！
